# Comparative efficacy and post-discharge adverse events of intranasal dexmedetomidine combined with different oral medications for procedural sedation in children: a randomized controlled trial

**DOI:** 10.3389/fphar.2026.1801603

**Published:** 2026-04-24

**Authors:** Haisu Li, Dan Zhou, Min Du, Ying Xu

**Affiliations:** 1 Department of Anesthesiology Children’s Hospital of Chongqing Medical University, National Clinical Research Center for Children and Adolescents’ Health and Diseases, Ministry of Education Key Laboratory of Child Development and Disorders, Chongqing Key Laboratory of Child Neurodevelopment and Cognitive Disorders, Chongqing, China; 2 Department of Anesthesiology, The Affiliated Dazu’s Hospital of Chongqing Medical University, Chongqing, China; 3 College of Pediatrics, Chongqing Medical University, Chongqing, China

**Keywords:** chloral hydrate, dexmedetomidine, midazolam, pediatric sedation, post-discharge adverse events

## Abstract

**Background:**

Combination sedation regimens are widely used in pediatric procedural sedation. However, the efficacy and incidence of post-discharge adverse events between intranasal dexmedetomidine combined with oral chloral hydrate and intranasal dexmedetomidine combined with oral midazolam remain unclear.

**Methods:**

This was a single-center, prospective, randomized controlled trial. A total of 180 children aged 1–6 years with American Society of Anesthesiologists physical status I–II who were scheduled for outpatient procedural sedation between December 2022 and October 2023 were enrolled and randomly assigned to receive intranasal dexmedetomidine combined with oral chloral hydrate (D+C group, n = 90) or intranasal dexmedetomidine combined with oral midazolam (D+M group, n = 90). The primary outcome was first-attempt sedation success. Secondary outcomes included the incidence of caregiver-reported post-discharge adverse events within 48 h, the incidence of peri-sedation adverse events, sedation onset time, procedure duration, recovery time, children’s medication acceptance, and parental satisfaction.

**Results:**

The first-attempt sedation success rate was significantly higher in the D+M group than in the D+C group (95.56% vs. 86.67%, P = 0.036). Sedation onset time was shorter in the D+C group (P < 0.001), whereas recovery time and peri-sedation adverse events were comparable between the two groups. Children’s medication acceptance and parental satisfaction were lower in the D+C group (P < 0.001). During the 0–48 h period after discharge, the D+C group had higher incidences of somnolence, ataxia, and any adverse event (all P < 0.05). Multivariable analysis confirmed that the D+M regimen was independently associated with lower risks of several post-discharge adverse events but a higher risk of behavioral changes. Phi coefficient analysis suggested clustering of adverse events, with neurologic symptoms tending to co-occur.

**Conclusion:**

Compared with the D+C regimen, the D+M regimen provides a higher first-attempt sedation success rate and fewer post-discharge adverse events, although attention to post-discharge behavioral changes remains necessary.

## Introduction

Children undergoing diagnostic evaluations or minor procedures are often required to remain still and cooperative. Owing to age-related cognitive and behavioral characteristics, a subset of children cannot maintain adequate immobility during examinations; therefore, procedural sedation has become an essential adjunct in pediatric outpatient and day-case care (Ramaiah and Bhananker, 2011; Merchant and Nadkarni, 2023). An optimal sedation regimen should provide sufficient conditions for the planned procedure while minimizing respiratory and hemodynamic compromise and facilitating smooth recovery. Discharge readiness assessment and caregiver education are likewise key components of sedation safety management (Coté and Wilson, 2006).

Needle-free sedation offers practical advantages in pediatric practice. Intranasal administration is noninvasive, easy to implement, and generally well accepted; moreover, it may partially bypass first-pass metabolism, which has supported its increasing use for imaging studies and brief procedures (de Rover et al., 2023; Haridoss et al., 2025; Tsze et al., 2025). Dexmedetomidine, a highly selective α_2_-adrenoceptor agonist, provides sedative and anxiolytic effects with relatively limited respiratory depression, and accumulating evidence supports the use of intranasal dexmedetomidine for sedation during pediatric examinations (Poonai et al., 2020; Kim et al., 2021; Chandrasekar et al., 2023). However, in scenarios requiring deeper levels of sedation, intranasal dexmedetomidine alone may be limited by a slower onset and/or insufficient sedation, and combination regimens are commonly adopted in clinical practice to improve sedation quality (Nie et al., 2024).

Oral chloral hydrate and oral midazolam remain two commonly used sedative agents in children. Systematic reviews indicate that chloral hydrate provides established sedative efficacy, although its adverse-event profile and tolerability warrant attention (Chen et al., 2019; Fong et al., 2021). Oral midazolam has also been shown to be feasible and effective in pediatric sedation, but inter-individual variability and behavioral responses should be considered (Cheng et al., 2020). Sedation guidelines emphasize that overall safety and patient experience depend not only on the choice of sedative agents but also on the standardization of peri-sedation process management, including pre-sedation assessment, fasting practices, recovery observation, and discharge guidance (Coté et al., 2019). With the expansion of day-case services, the evaluation and management of sedation outcomes should not be confined to in-hospital monitoring (McQueen et al., 2009). Previous studies have reported caregiver-observed post-discharge events—such as somnolence, gait instability/ataxia, sleep pattern disturbance, nausea/vomiting, reduced appetite, and behavioral changes—which may impair recovery quality and increase caregiver burden (Costa et al., 2012; Alha et al., 2022).

However, prospective comparative data regarding the success rate and post-discharge adverse events of intranasal dexmedetomidine combined with different oral medications for pediatric procedural sedation remain limited. This randomized controlled trial compared the success rates and post-discharge adverse events of intranasal dexmedetomidine combined with oral midazolam versus chloral hydrate for pediatric sedation, aiming to provide evidence for clinical protocol selection and optimization of post-procedural management.

## Materials and methods

### Study design and ethics

This single-center, prospective, double-blind randomized controlled trial was conducted in accordance with the CONSORT statement (Schulz et al., 2010). The study protocol was approved by the Ethics Committee of Children’s Hospital of Chongqing Medical University (Approval No. (2022) Scientific Research Ethics Review No. 508) and was prospectively registered with the Chinese Clinical Trial Registry (ChiCTR2300067629). The study was performed in compliance with the Declaration of Helsinki. Written informed consent was obtained from the legal guardians of all enrolled children. The trial was conducted at Children’s Hospital of Chongqing Medical University between December 2022 and October 2023.

### Participants

Children who need to remain still for diagnostic or therapeutic procedures, or who are unable to cooperate due to fear or anxiety, receive outpatient procedural sedation. Eligibility for study enrollment is assessed based on predefined inclusion and exclusion criteria. Inclusion criteria were: outpatient sedation at our institution during the study period, American Society of Anesthesiologists (ASA) physical status I–II, age 1–6 years, and provision of written informed consent by a legal guardian. Exclusion criteria were: known allergy to midazolam, dexmedetomidine, or other study-related medications; use of sedative-hypnotic medication within 24 h or a history of sedation failure; severe cardiovascular, pulmonary, or neurologic disease; anticipated difficulty with intranasal or oral drug administration; or refusal by the guardian to participate.

### Randomization and blinding

A computer-generated random sequence was used to allocate participants in a 1:1 ratio to the intranasal dexmedetomidine combined with oral midazolam group (D+M) or the intranasal dexmedetomidine combined with oral chloral hydrate group (D+C). Allocation was implemented using sealed opaque envelopes. The child’s guardians and the investigators involved in data analysis remained blinded to group assignment throughout the study.

### Sedation protocol and outcome assessment

All sedations were managed by anesthesiologists with more than 10 years of experience in pediatric sedation. Guardians received standardized fasting instructions before the examination, and a pre-sedation assessment was completed on the day of the procedure. In the D+M group, dexmedetomidine (Youbituo®, 100 μg/mL; Yangzijiang Pharmaceutical Group, Jiangsu, China) was administered intranasally at 2 μg/kg using a 1 mL needleless syringe, with the child in the supine position and the dose divided between both nostrils. Oral midazolam solution (Xiao’erjing®, 20 mg/mL; Yichang Humanwell Pharmaceutical Co., Ltd., Hubei, China) was then given without dilution at 0.25 mg/kg. In the D+C group, the same intranasal dexmedetomidine administration was followed by oral chloral hydrate syrup at 30 mg/kg.

Sedation depth was assessed using the Ramsay Sedation Scale (RSS) (Ramsay et al., 1974; Li and Lei, 2025). Optimal sedation was defined as no response to verbal stimulation with a purposeful response to tactile or painful stimulation (RSS 4–5, Supplementary Table S1). Sedation failure was defined as inability to complete the examination due to movement. When sedation failure occurred, the attending anesthesiologist determined whether inhaled sevoflurane would be used to deepen sedation to complete the examination. Recovery before discharge was assessed using the modified Aldrete score (activity, respiration, circulation, consciousness, and oxygen saturation; 0–2 points per domain), and a total score ≥9 was considered discharge-eligible (Supplementary Table S2) (Aldrete and Kroulik, 1970; Aldrete, 1995). Children’s acceptance of medication during administration was rated using a 5-point Likert scale (1 = worst, 5 = best) based on behavioral observation (Supplementary Table S3). Parental satisfaction was also rated on a 5-point Likert scale (1 = worst, 5 = best) (Supplementary Table S4).

During sedation and recovery, continuous monitoring was performed and peri-sedation adverse events were systematically recorded. Bradycardia was defined as heart rate below the lower limit of normal for age during sleep or a decrease of ≥20% from baseline. Respiratory depression was defined as respiratory rate below the normal range for age or reduced respiratory effort requiring stimulation, repositioning, or assisted ventilation to maintain effective ventilation. Hypoxemia was defined as SpO_2_ <90% lasting >10 s. Delayed recovery was defined as failure to reach an Aldrete score ≥9 more than 2 h after the end of sedation.

Post-discharge adverse events were collected via a questionnaire delivered through the sedation system after discharge. Caregivers provided free-text descriptions of symptoms. Two experienced anesthesiologists independently reviewed the responses and extracted adverse event occurrences; disagreements were adjudicated by a third anesthesiologist.

### Outcome measures

The primary outcome was the success rate of first-attempt sedation. Secondary outcomes included the incidence of caregiver-reported post-discharge adverse events within 48 h after discharge, the incidence of peri-sedation adverse events, sedation onset time, procedure duration, recovery time, children’s medication acceptance, and parental satisfaction. The definition of post-discharge adverse events is provided in Supplementary Table S5.

### Sample size and statistical analysis

Sample size estimation was based on pilot study data, which showed a first-attempt sedation success rate of 96.5% in the D+M group and 83.4% in the D+C group. With 80% power, a two-sided α level of 0.05 (0.025 per side), and an anticipated dropout rate of 10%, the required sample size was calculated as 180 participants.

Statistical analysis was performed as follows. The Shapiro–Wilk test was used to assess the normality of continuous variables. Continuous data are presented as mean ± standard deviation (SD) or median (interquartile range [IQR]) and were compared using the t-test or Mann–Whitney U test, as appropriate. Categorical variables are presented as counts (percentages) and were compared using the chi-square test or Fisher’s exact test. To evaluate independent associations between the sedation regimen and post-discharge adverse events, multivariable logistic regression was performed. Candidate covariates were selected based on univariable screening and clinical relevance, and results are reported as odds ratios (ORs) with 95% confidence intervals (CIs) and P values. Co-occurrence patterns among adverse events were assessed using Phi coefficients (φ) for binary variables and visualized as a heatmap. A P value < 0.05 was considered statistically significant. All analyses were performed using R software (version 4.3.2).

## Results

### Baseline characteristics

**FIGURE 1:**
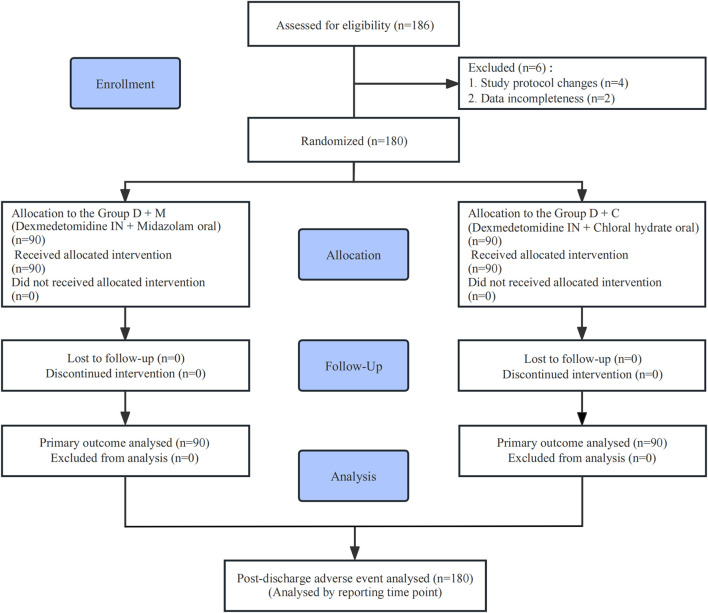
CONSORT flow diagram of the study. Abbreviations: CONSORT, Consolidated Standards of Reporting Trials; Group D+M, received intranasal dexmedetomidine combined with oral midazolam; Group D+C, received intranasal dexmedetomidine combined with oral chloral hydrate.

Among the 186 children screened, 4 were excluded due to adjustments in the sedation protocol, and 2 were excluded due to incomplete data. A total of 180 children were ultimately randomized and included in the analysis; all participants received the allocated intervention, and no loss to follow-up occurred (Figure 1). Baseline characteristics and examination types were comparable between the two groups (all P > 0.05; Table 1). Examination types included pulmonary function tests (52.22%), magnetic resonance imaging (2.78%), electroencephalography (5.56%), hearing screening (6.11%), electrocardiography (10.56%), specialized ultrasonography (18.33%), and computed tomography (4.44%).

**TABLE 1:** Baseline characteristics.

Variables	Total (n = 180)	Group D+C (n = 90)	Group D+M (n = 90)
Sex, n (%)			
Male	92 (51.11)	49 (54.44)	43 (47.78)
Female	88 (48.89)	41 (45.56)	47 (52.22)
Age (months), M (Q_1_, Q_3_)	31.00 (21.00, 41.00)	31.00 (22.25, 41.00)	29.50 (21.00, 39.75)
Weight (kg), M (Q_1_, Q_3_)	13.00 (11.30, 15.00)	13.00 (11.50, 15.00)	13.00 (11.07, 14.50)
Body temperature (°C), M (Q_1_, Q_3_)	36.60 (36.40, 36.73)	36.60 (36.42, 36.70)	36.60 (36.40, 36.80)
ASA, n (%)			
I	19 (10.56)	9 (10.00)	10 (11.11)
II	161 (89.44)	81 (90.00)	80 (88.89)
Fasting time (h), M (Q_1_, Q_3_)	2.51 (1.92, 4.35)	2.91 (1.77, 5.61)	2.41 (1.96, 3.98)
Previous sedation history, n (%)	28 (15.56)	13 (14.44)	15 (16.67)
Primary diagnosis, n (%)			
Routine health examination	8 (4.44)	4 (4.44)	4 (4.44)
Respiratory system disorders	102 (56.67)	49 (54.44)	53 (58.89)
Neurological disorders	17 (9.44)	12 (13.33)	5 (5.56)
Digestive system disorders	6 (3.33)	2 (2.22)	4 (4.44)
Cardiovascular disorders	35 (19.44)	15 (16.67)	20 (22.22)
Sensory system disorders	12 (6.67)	8 (8.89)	4 (4.44)
Examination types, n (%)			
Pulmonary function test	94 (52.22)	44 (48.89)	50 (55.56)
Magnetic resonance imaging (MRI)	5 (2.78)	3 (3.33)	2 (2.22)
Electroencephalography (EEG)	10 (5.56)	8 (8.89)	2 (2.22)
Hearing screening	11 (6.11)	7 (7.78)	4 (4.44)
Electrocardiography (ECG)	19 (10.56)	8 (8.89)	11 (12.22)
Specialized ultrasonography	33 (18.33)	16 (17.78)	17 (18.89)
Computed tomography (CT)	8 (4.44)	4 (4.44)	4 (4.44)

Data are presented as median (interquartile range) or number (%), as appropriate.

Abbreviations: Group D+C received intranasal dexmedetomidine combined with oral chloral hydrate, and Group D+M received intranasal dexmedetomidine combined with oral midazolam.

### Peri-sedation outcomes

Compared with the D+M group, the D+C group had a lower first-attempt sedation success rate (86.67% vs. 95.56%, P = 0.036), a shorter sedation onset time (median [IQR]: 18.50 [15.00, 23.00] minutes vs. 23.00 [18.00, 30.00] minutes; P < 0.001), and showed no statistically significant difference in recovery time (30.50 [20.25, 37.00] minutes vs. 26.00 [16.25, 34.00] minutes; P = 0.052). Peri-sedation adverse events (including bradycardia, respiratory depression, hypoxemia, nausea/vomiting, and delayed recovery) did not differ between the groups (P > 0.05). In addition, children’s medication acceptance and parental satisfaction scores were higher in the D+M group than in the D+C group (P < 0.001; Table 2).

**TABLE 2:** Peri-sedation outcomes.

Variables	Total (n = 180)	Group D+C (n = 90)	Group D+M (n = 90)	*P*
First-attempt sedation success, n (%)	164 (91.11)	78 (86.67)	86 (95.56)	0.036
Sedation onset time (min), M (Q_1_, Q_3_)	20.50 (15.75, 26.00)	18.50 (15.00, 23.00)	23.00 (18.00, 30.00)	<0.001
Procedure duration (min), M (Q_1_, Q_3_)	68.00 (63.00, 80.00)	68.00 (64.00, 77.75)	68.00 (62.25, 80.75)	0.544
Recovery time (min), M (Q_1_, Q_3_)	27.00 (19.00, 35.00)	30.50 (20.25, 37.00)	26.00 (16.25, 34.00)	0.052
Bradycardia, n (%)	19 (10.56)	12 (13.33)	7 (7.78)	0.225
Respiratory depression, n (%)	10 (5.56)	6 (6.67)	4 (4.44)	0.515
Hypoxemia, n (%)	6 (3.33)	4 (4.44)	2 (2.22)	0.678
Nausea and vomiting, n (%)	5 (2.78)	4 (4.44)	1 (1.11)	0.364
Delayed recovery	1 (0.56)	1 (1.11)	0 (0.00)	1.000
Children’s medication acceptance, M (Q_1_, Q_3_)	4.00 (4.00, 4.00)	4.00 (3.00, 4.00)	4.00 (4.00, 5.00)	<0.001
Parental satisfaction, M (Q_1_, Q_3_)	4.00 (4.00, 4.00)	4.00 (4.00, 4.00)	4.00 (4.00, 5.00)	<0.001

Data are presented as median (interquartile range) or number (%), as appropriate.

Abbreviations: Group D+C received intranasal dexmedetomidine combined with oral chloral hydrate, and Group D+M received intranasal dexmedetomidine combined with oral midazolam.

### Post-discharge adverse events

Post-discharge events were analyzed by time window (Table 3). During 0–24 h after discharge, the D+C group had higher incidences of somnolence (72.97% vs. 46.03%; P = 0.009), decreased appetite (35.14% vs. 15.87%; P = 0.027), ataxia (35.14% vs. 14.29%; P = 0.015), and any adverse event (86.49% vs. 68.25%; P = 0.042). Behavioral changes were more frequent in the D+M group (33.33% vs. 13.51%; P = 0.029). During 24–48 h, somnolence (64.15% vs. 37.04%; P = 0.021), ataxia (26.42% vs. 7.41%; P = 0.044), and any adverse event (81.13% vs. 59.26%; P = 0.036) remained higher in the D+C group.

**TABLE 3:** Post-discharge adverse events by time window.

Variables	24 h post-discharge				24–48 h post-discharge			
	Total (n = 100)	Group D+C (n = 37)	Group D+M (n = 63)	*P*	Total (n = 80)	Group D+C (n = 53)	Group D+M (n = 27)	*P*
Somnolence, n (%)	56 (56.00)	27 (72.97)	29 (46.03)	0.009	44 (55.00)	34 (64.15)	10 (37.04)	0.021
Nausea and vomiting, n (%)	9 (9.00)	6 (16.22)	3 (4.76)	0.116	6 (7.50)	5 (9.43)	1 (3.70)	0.637
Decreased appetite, n (%)	23 (23.00)	13 (35.14)	10 (15.87)	0.027	23 (28.75)	18 (33.96)	5 (18.52)	0.149
Diarrhea, n (%)	2 (2.00)	1 (2.70)	1 (1.59)	1.000	6 (7.50)	5 (9.43)	1 (3.70)	0.637
Sleep pattern disturbance, n (%)	25 (25.00)	13 (35.14)	12 (19.05)	0.073	23 (28.75)	18 (33.96)	5 (18.52)	0.149
Behavioral changes, n (%)	26 (26.00)	5 (13.51)	21 (33.33)	0.029	16 (20.00)	10 (18.87)	6 (22.22)	0.723
Ataxia, n (%)	22 (22.00)	13 (35.14)	9 (14.29)	0.015	16 (20.00)	14 (26.42)	2 (7.41)	0.044
Respiratory depression, n (%)	4 (4.00)	2 (5.41)	2 (3.17)	0.983	1 (1.25)	0 (0.00)	1 (3.70)	0.337
Dizziness, n (%)	5 (5.00)	4 (10.81)	1 (1.59)	0.117	3 (3.75)	2 (3.77)	1 (3.70)	1.000
Cough, n (%)	7 (7.00)	5 (13.51)	2 (3.17)	0.121	7 (8.75)	6 (11.32)	1 (3.70)	0.470
Return visit, n (%)	4 (4.00)	2 (5.41)	2 (3.17)	0.983	1 (1.25)	1 (1.89)	0 (0.00)	1.000
Any adverse event, n (%)	75 (75.00)	32 (86.49)	43 (68.25)	0.042	59 (73.75)	43 (81.13)	16 (59.26)	0.036

Data are presented as median (interquartile range) or number (%), as appropriate.

Abbreviations: Group D+C received intranasal dexmedetomidine combined with oral chloral hydrate, and Group D+M received intranasal dexmedetomidine combined with oral midazolam.

### Multivariable analyses and event clustering

**FIGURE 2:**
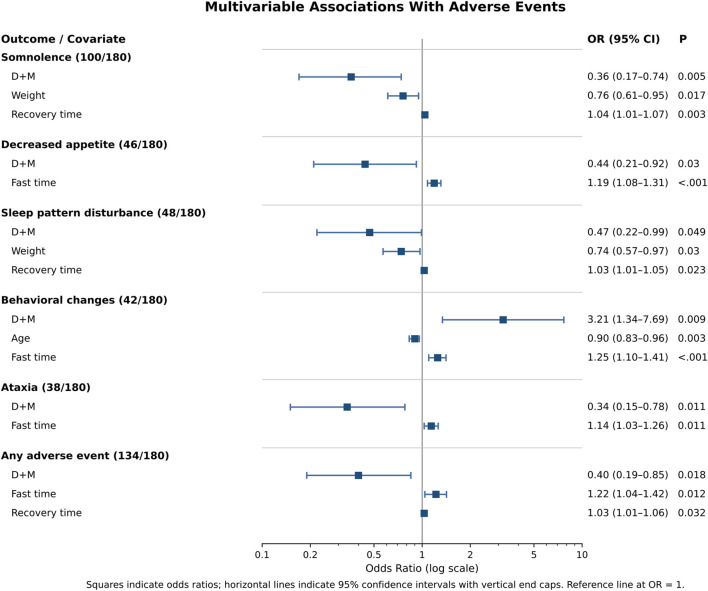
Multivariable analysis of post-discharge adverse events. Abbreviations: Group D+M, received intranasal dexmedetomidine combined with oral midazolam.

**FIGURE 3:**
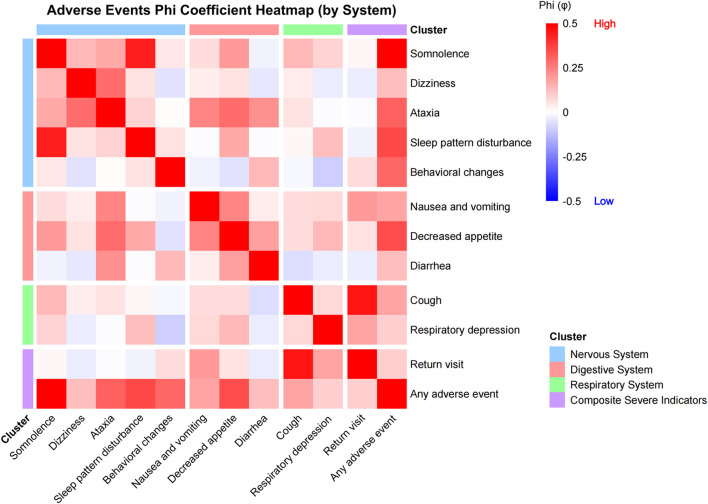
Co-occurrence of post-discharge adverse events. Note: Calculation of Phi (φ) coefficients for pairwise adverse event associations based on binary variables, grouped and displayed by system. Red indicates a positive correlation, blue indicates a negative correlation, and white indicates no significant correlation; color depth represents the strength of the correlation.

In multivariable logistic regression, compared with the D+C regimen, the D+M regimen was associated with lower odds of somnolence (OR (95% CI): 0.36 (0.17–0.74); P = 0.005), decreased appetite (0.44 (0.21–0.92); P = 0.030), sleep pattern disturbance (0.47 (0.22–0.99); P = 0.049), ataxia (0.34 (0.15–0.78); P = 0.011), and any adverse event (0.40 (0.19–0.85); P = 0.018), but higher odds of behavioral changes (3.21 (1.34–7.69); P = 0.009) (Figure 2). Longer fasting and recovery times were generally associated with increased risks of post-discharge adverse events, whereas higher body weight was associated with lower risks of somnolence and sleep pattern disturbance (Figure 2). Phi-coefficient analyses suggested clustering of adverse events, with neurologic symptoms more likely to co-occur (Figure 3).

## Discussion

This randomized controlled trial compared the first-attempt sedation success rate and post-discharge adverse events following pediatric outpatient sedation with intranasal dexmedetomidine co-administered with different oral agents. Overall, the D+M regimen achieved a higher first-attempt sedation success rate and was associated with fewer adverse events during 0–48 h after discharge. The D+M regimen was associated with lower incidences of somnolence, ataxia, and any adverse event, but a higher incidence of behavioral changes. Multivariable models indicated that fasting duration and recovery time were independently associated with several post-discharge adverse events. Correlation analyses further suggested clustering of adverse events, with neurologic manifestations more likely to co-occur.

The observed higher first-attempt sedation success rate with the D+M regimen compared to the D+C regimen is consistent with existing evidence indicating that the dexmedetomidine-midazolam combination provides more reliable procedural sedation than other non-IV alternatives, as supported by a recent meta-analysis demonstrating its superior success rates across various diagnostic settings (Nie et al., 2024). However, the larger efficacy difference observed in this trial compared to some observational studies on dexmedetomidine-chloral hydrate combinations—where success rates often appeared comparable between regimens—may stem from variations in patient populations, procedural requirements, success criteria, or administration protocols (Wang et al., 2024). Pharmacologically, midazolam enhances anxiolysis and amnesia, promoting cooperation during positioning and reducing early failure due to agitation, whereas chloral hydrate exhibits unpredictable absorption, is irritant and unpalatable, factors that can lead to incomplete dosing through spitting or vomiting and may thereby compromise first-attempt success in outpatient practice (Nie et al., 2024). This aligns with earlier reviews favoring intranasal dexmedetomidine over chloral hydrate for imaging sedation, underscoring that regimen composition and drug delivery route can substantially influence initial sedation outcomes (Lyu et al., 2022).

The safety margin of pediatric sedation is not determined solely by in-hospital monitoring. The AAP/AAPD guideline recommends discharge only after recovery to near-baseline status and emphasizes clear caregiver instructions for post-discharge observation and management (Coté and Wilson, 2006). By extending outcomes beyond discharge, our findings support that comparable in-hospital indicators do not necessarily translate into similar post-discharge risk, underscoring the importance of discharge planning and caregiver education.

Somnolence and ataxia occurred more frequently after discharge in the D+C group, consistent with prior summaries of the adverse event profile of chloral hydrate (Chen et al., 2019; Fong et al., 2021). Follow-up data from imaging sedation have also shown that chloral hydrate–related symptoms may persist for hours after discharge (Alha et al., 2022). In addition, reports of severe toxicity events associated with outpatient chloral hydrate use highlight that its safety margin is highly dependent on standardized processes and effective risk communication (Malviya et al., 2000). The higher frequency of reduced appetite in the D+C group in our cohort suggests that, beyond regimen selection, post-discharge guidance regarding diet and activity restrictions remains necessary to reduce caregiver burden and potential safety risks.

The D+M regimen showed a protective association for multiple post-discharge adverse events. This finding aligns with evidence from systematic evaluations of dexmedetomidine combined with midazolam for pediatric procedural sedation (Nie et al., 2024). From a pharmacologic perspective, the pharmacokinetic profile of midazolam is relatively predictable, which may partly explain the observed differences in recovery-phase manifestations between regimens (Cheng et al., 2020). Notably, clinically significant respiratory events during the peri-sedation period were infrequent and similar between groups, suggesting that the between-group differences were more apparent after discharge and during home recovery, where residual effects are more readily perceived by caregivers.

Behavioral changes were more common in the D+M group and represent an important additional observation. Paradoxical reactions to midazolam, including irritability, inconsolable crying, aggression, or behavioral dysregulation, have been described and appear to vary by age and individual susceptibility (Coté et al., 2000; Moon, 2013). Accordingly, discharge counseling should address the possibility of post-discharge behavioral fluctuations and provide practical home-management strategies to reduce caregiver distress and avoid unnecessary return visits.

Fasting duration and recovery time showed stable associations across models. Prolonged fasting is linked to thirst, hunger, and irritability and has become a key target in optimizing peri-sedation workflows in children (Coté et al., 2019; Dahmani et al., 2014). Prolonged recovery time may reflect more prominent residual sedation, increasing the likelihood of post-discharge somnolence or sleep pattern disturbance. Behavioral outcomes may also be influenced by sleep state and circadian factors: reviews have associated pediatric emergence agitation with sleep-related factors (Wei et al., 2024), recent evidence suggests diurnal variation may affect emergence behavior (Malviya et al., 1997), and sleep quality has been linked to emergence agitation risk (Kuratani and Oi, 2008). Sleep deprivation, which is sometimes used as an adjunct to facilitate sedation, has inconsistent benefit and may carry behavioral trade-offs; prior work indicates it does not necessarily improve the success rate of chloral hydrate sedation (Voepel-Lewis et al., 2003). These considerations suggest that reducing post-discharge adverse events requires not only medication selection but also process optimization, including waiting time management, fasting policies, and the quality of recovery observation.

The phi-coefficient heatmap indicates that adverse events often occur in combination rather than in isolation, with somnolence, ataxia, and sleep disturbance tending to co-occur. Although these behavioral changes are typically transient, mild, and rarely require intervention, they still warrant attention. Post-discharge safety measures should therefore prioritize fall prevention, restriction of vigorous activity, and monitoring of mental status and sleep, along with clear caregiver thresholds for contacting the hospital or returning for evaluation, consistent with prior emphasis on identifying post-discharge events and follow-up needs (Costa et al., 2012).

Several limitations of this study should be acknowledged. As a single-center study, its generalizability may be limited. Due to differences in the taste and dosage volume of oral midazolam and chloral hydrate, complete blinding of caregivers may not have been fully achieved. Although a difference in first-attempt sedation success was observed between the two groups, this outcome could have been influenced by the definition of sedation success, medication adherence issues (e.g., refusal/incomplete ingestion or vomiting), and procedural (notably, the high proportion of pulmonary function tests) or environmental factors. Post-discharge adverse events were reported by caregivers as free-text descriptions of children’s symptoms, without structured assessment. Although these symptom descriptions were reviewed by anesthesiologists, reporting variability may still exist. To capture the temporal trajectory of adverse events, caregivers were instructed to complete questionnaires immediately upon the occurrence of any event; however, reporting across different time windows resulted in varying numbers of respondents across periods, which may affect the representativeness of the findings. Furthermore, as the reasons for sedation failure were not further stratified, and sevoflurane inhalation was used as a rescue intervention after first-attempt failure, this may have influenced post-discharge outcomes. These findings therefore require cautious interpretation. Future multicenter studies are still needed to refine post-discharge follow-up tools and to develop risk-stratification strategies for pediatric outpatient sedation.

## Conclusion

In pediatric sedation, compared with the chloral hydrate regimen, intranasal dexmedetomidine combined with oral midazolam demonstrated higher sedation success rates and fewer overall adverse events within 48 h post-discharge, particularly reducing residual sedation-related events such as somnolence, sleep pattern disturbance, and ataxia. However, this regimen may be associated with an increased risk of behavioral changes, necessitating targeted caregiver education and follow-up.

## Data Availability

The datasets supporting the conclusions of this study are available from the corresponding authors upon reasonable request.
