# Supplementary material for: Comparative efficacy and post-discharge adverse events of intranasal dexmedetomidine combined with different oral medications for procedural sedation in children: a randomized controlled trial
Source: Front Pharmacol. 2026 Apr 24;17:1801603. doi: 10.3389/fphar.2026.1801603 (PMC13154392; doi:10.3389/fphar.2026.1801603)
Supplement: Supplementary file 1 [file Table1.docx]

Supplementary Table 1. Ramsay Sedation Scale (RSS) and clinical descriptions.

| Score | Clinical description |
| --- | --- |
| 1 | Anxious, agitated, or restless |
| 2 | Cooperative, oriented, and tranquil |
| 3 | Responds to verbal commands only |
| 4 | Brisk response to light glabellar tap or loud auditory stimulus |
| 5 | Sluggish response to light glabellar tap or loud auditory stimulus |
| 6 | No response to stimulus |

Supplementary Table 2. Aldrete score components and scoring criteria.

| Domain | Score 0 | Score 1 | Score 2 |
| --- | --- | --- | --- |
| Activity | Unable to move extremities | Moves 2 extremities | Moves all extremities |
| Respiration | Apnea | Dyspnea or shallow breathing | Able to breathe deeply and cough |
| Circulation | BP ±50% of pre-anesthetic level | BP ±20–50% of baseline | BP ±20% of baseline |
| Consciousness | Not responding | Arousable on calling | Fully awake |
| Oxygen saturation | SpO₂ <90% with oxygen | SpO₂ >90% with oxygen | SpO₂ >92% on room air |

Supplementary Table 3. Five-point scale for children’s medication acceptance during administration.

| Score | Definition |
| --- | --- |
| 1 | Severe resistance, crying, or refusal |
| 2 | Marked resistance, poor cooperation |
| 3 | Moderate acceptance with intermittent resistance |
| 4 | Good acceptance with minimal resistance |
| 5 | Excellent acceptance, calm and cooperative |

Supplementary Table 4. Five-point Likert scale for parental satisfaction.

| Score | Definition |
| --- | --- |
| 1 | Very dissatisfied |
| 2 | Dissatisfied |
| 3 | Neutral |
| 4 | Satisfied |
| 5 | Very satisfied |

Supplementary Table 5. Types and Definitions of Post-Discharge Adverse Events in Pediatric Patients.

| Post-Discharge Adverse Events | Definition (occurring within 48 hours) |
| --- | --- |
| Somnolence | Unusual drowsiness or prolonged sleepiness compared with the child’s usual state after discharge. |
| Nausea and vomiting | Any complaint of nausea and/or any episode of vomiting after discharge. |
| Decreased appetite | Reduced oral intake or unwillingness to eat compared with usual intake after discharge. |
| Diarrhea | Loose or frequent stools after discharge. |
| Sleep pattern disturbance | Difficulty falling asleep, frequent awakening, excessive sleep, or an altered sleep–wake pattern compared with usual. |
| Behavioral changes | New or worsened irritability, inconsolable crying, agitation, aggression, withdrawal, or other behavior different from baseline. |
| Ataxia | Unsteady gait, impaired coordination, or need for extra assistance when standing or walking after discharge. |
| Respiratory depression | Slow, shallow, labored, or paused breathing, cyanosis, or any breathing problem requiring stimulation, oxygen, or medical attention. |
| Dizziness | Complaint of dizziness/vertigo or caregiver-observed lightheadedness or unsteadiness after discharge. |
| Cough | New-onset or worsened cough after discharge. |
| Return visit | Any unscheduled medical revisit after discharge, including clinic, emergency department, or hospital visit. |
| Any adverse event | Occurrence of any of the above post-discharge adverse events. |
